# *In situ* fabrication of high-performance Ni-GDC-nanocube core-shell anode for low-temperature solid-oxide fuel cells

**DOI:** 10.1038/srep17433

**Published:** 2015-11-30

**Authors:** Kazuhiro Yamamoto, Nan Qiu, Satoshi Ohara

**Affiliations:** 1 Joining and Welding Research Institute, Osaka University, 11-1 Mihogaoka, Ibaraki, Osaka 567-0047, Japan

## Abstract

A core–shell anode consisting of nickel–gadolinium-doped-ceria (Ni–GDC) nanocubes was directly fabricated by a chemical process in a solution containing a nickel source and GDC nanocubes covered with highly reactive {001} facets. The cermet anode effectively generated a Ni metal framework even at 500 °C with the growth of the Ni spheres. Anode fabrication at such a low temperature without any sintering could insert a finely nanostructured layer close to the interface between the electrolyte and the anode. The maximum power density of the attractive anode was 97 mW cm^–2^, which is higher than that of a conventional NiO–GDC anode prepared by an aerosol process at 55 mW cm^–2^ and 600 °C, followed by sintering at 1300 °C. Furthermore, the macro- and microstructure of the Ni–GDC-nanocube anode were preserved before and after the power-generation test at 700 °C. Especially, the reactive {001} facets were stabled even after generation test, which served to reduce the activation energy for fuel oxidation successfully.

A cermet consisting of the nickel–gadolinium-doped-ceria (Ni–GDC) composite has generated much interest as an attractive anode material for low-temperature solid-oxide fuel cells (SOFCs) because its oxygen ionic conductivity is higher than that of conventional nickel–yttrium-stabilized zirconium (Ni–YSZ) cermet anodes at operating temperatures below 700 °C. The oxygen ionic conductivity of GDC (Ce_0.9_Gd_0.1_O_1.95_) has been reported to be approximately 0.01, 0.025, and 0.054 S cm^–1^ at 500, 600, and 700 °C, respectively^1^. In other words, the diffusion of oxygen ions is seriously inhibited by decreases in the operating temperature. Therefore, the effective reaction sites for power generation at low temperatures (500–600 °C) in an anode are confined to areas close to the interface between the solid electrolyte and the anode. Some researchers have reported that increasing the triple-phase boundary (TPB) effectively introduced the nanostructures to the electrolyte–anode interface for low-temperature operation^2^,^3^,^4^,^5^. In general, a conventional SOFC anode is fabricated as follows. First, NiO and an oxygen-ion conductor powder are mixed with a binder, after which the anode paste is coated on the electrolyte. The anode is then sintered at ∼1300 °C to form the necking structure of anode materials for power generation and collection. Finally, NiO is reduced to metallic nickel during the power-generation test^6^,^7^. The second step—fabricating the necking structure by high-temperature sintering—is regarded as the essential process. However, it prevents the insertion of fine nanostructures near the electrolyte–anode interface because of coarsening of the anode materials. Thus, a new process without high-temperature sintering would allow insertion of fine nanostructures, which would help realize the fabrication of anodes that remain stable before and after the power-generation test.

We have reported organic-ligand-assisted hydrothermal synthesis of ceria and GDC nanocubes covered with highly reactive {001} crystal facets^8^,^9^,^10^,^11^, which are considered an attractive catalyst and a good oxygen-ion conductor, respectively. In the study reported here, we examined direct fabrication of a Ni–GDC-nanocube cermet anode by chemical reaction in a solution. The anode material had a unique core–shell structure composed of 100–200-nm spherical Ni-metal particles covered with 10 nm GDC nanocubes. After screen printing of the anode paste on the solid electrolyte, a power-generation test was performed directly without any sintering. Through the growth of just a few crystals of Ni spheres, a Ni metal framework for electrical power collection was successfully fabricated during a power-generation test at 500 °C. In addition, a vastly enlarged TPB could be inserted near the electrolyte–anode interface without sintering owing to the 10 nm dimensions of fine GDC nanocubes. The anode macrostructure, consisting of individual large spherical shapes and a self-fabricated Ni metal framework, provided superior porosity and electrical-power-collection pathways. Meanwhile, the reactive nanostructure was attained by controlling the particle size and crystal plane of the oxygen ionic conductor. Thus, we could obtain the desired electrode structure through this new strategy for *in situ* anode fabrication without any sintering. The novel fabrication process of a high-performance core–shell Ni–GDC-nanocube anode and its excellent power-generation property are presented here.

## Results

All samples prepared by the chemical-reduction method showed multiphase diffraction patterns from face-centered cubic (fcc) nickel metal and CeO_2_ with a cubic fluorite structure (Figure S1). The diffraction peak intensities changed systematically with the initial ratios of Ni to GDC (v:v).

**Figure 1:**
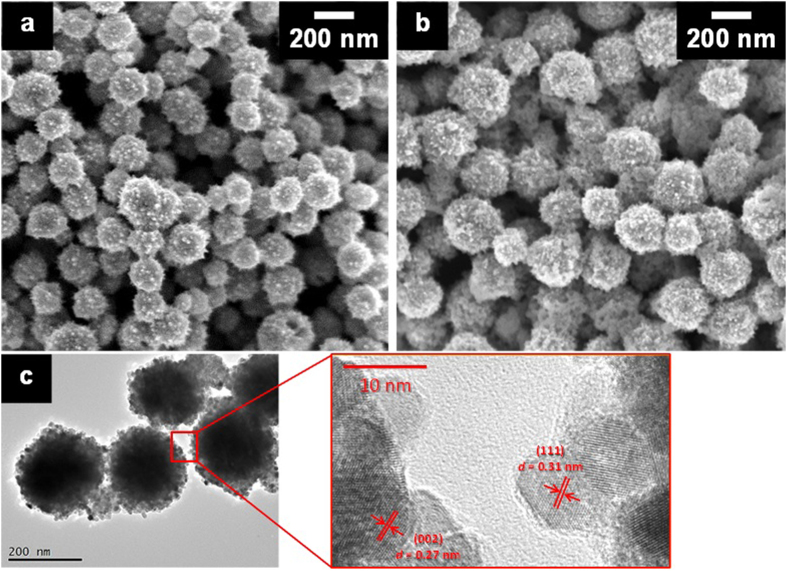
Microscope images of GDC samples. GDC samples prepared by the chemical-reduction method (**a**) without GDC nanocube dispersion and (**b**) with 2.66 mL of a GDC dispersion (Ni:GDC = 65:35). (**c**) (Left) TEM and (Right) HR-TEM images of (**b**).

As shown in Fig. 1a, the sample obtained by chemical reduction in a solution without GDC dispersion (metallic Ni sample) had a spherical morphology, with particle diameters of 100–200 nm. On the other hand, the Ni–GDC-nanocube composite sample (Ni:GDC = 65:35) also had a spherical morphology with particle diameters of 200–300 nm, which are slightly larger than the diameters of the metallic Ni particles (Fig. 1b). Furthermore, the microstructure of the Ni–GDC-nanocube composite sample was confirmed by detailed observation with transmission electron microscopy (TEM). In Fig. 1c, the spherical composite particles with diameters of 200–300 nm had a very fine nanostructure on the surface, and high-resolution (HR)-TEM observation identified that the nanostructure corresponded to the characteristic (002) and (111) lattice fringes of CeO_2_. These results indicate that the Ni–GDC-nanocube composite samples had a core–shell morphology, which consisted of a metallic Ni core covered with GDC-nanocube fine particles measuring 10 nm in size. Many researchers have reported synthesis of metallic Ni particles by the chemical-reduction method; the reaction mechanism is summarized as follows^12^,^13^,^14^:




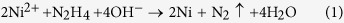




Initially, the Ni^2+^ ions generate a light-blue complex [Ni(N_2_H_4_)_2_]Cl_2_ with excess hydrazine and the complex precipitates. The [Ni(N_2_H_4_)_2_]Cl_2_ complex is immediately reduced by adding a NaOH solution. Very fine metal Ni particles can then easily aggregate owing to van der Waals forces and the magnetism, and such an agglomeration can lead to the crystal growth of metallic Ni particles by Ostwald ripening^11^. In our fabrication process, the [Ni(N_2_H_4_)_2_]Cl_2_ complex and GDC nanocubes were co-precipitated with hydrazine. During the agglomeration and Ostwald ripening, metallic Ni gathered towards the interior as the spherical core and grew to a large sphere measuring 100 to 200 nm in size, while the GDC nanocubes were pushed towards the exterior to cover the metallic Ni sphere.

**Figure 2:**
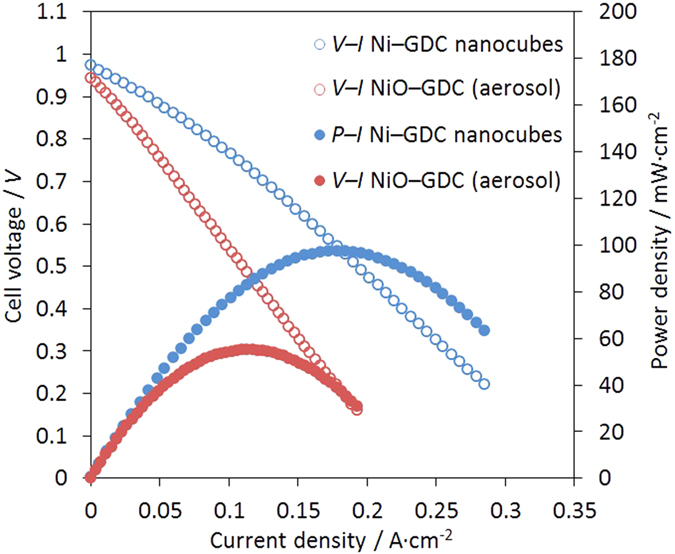
*V–I* and *P–I* curves of single cells. (**a**) Ni–GDC-nanocube anode (Ni:GDC = 65:35) and (**b**) NiO-GDC anode prepared by aerosol process and sintering at 1300 °C. The cell-performance tests were performed at 600 °C.

After screen printing of the Ni–GDC-nanocube (Ni:GDC = 65:35) paste, the solid-oxide single fuel cells were directly examined to investigate the power-generation properties at temperatures between 500 and 700 °C. The voltage–current (*V*–*I*) and power–current (*P*–*I*) curves are shown in Figure S2. Power generation was detected even at 500 °C, which indicates that the anode structure for electrical-power generation and collection was successfully fabricated without any sintering of the anode material. Furthermore, higher power densities were obtained at higher operating temperatures. The maximum power densities were 25, 51, 97, 158, and 224 mW·cm^–2^ at 500, 550, 600, 650, and 700 °C, respectively. When the power-generation property at 600 °C was compared with that of a NiO-GDC anode that was prepared by an aerosol process followed by sintering at 1300 °C^15^,^16^,^17^, the Ni–GDC-nanocube (Ni:GDC = 65:35) anode showed a maximum power density that was 1.8 times higher (Fig 2). In addition, the Ni–GDC-nanocube cermet anode had very high performance that was comparable to that of a NiO–GDC-nanocube compo site anode fabricated by an aerosol process and sintering at 1100 °C^11^.

**Figure 3:**
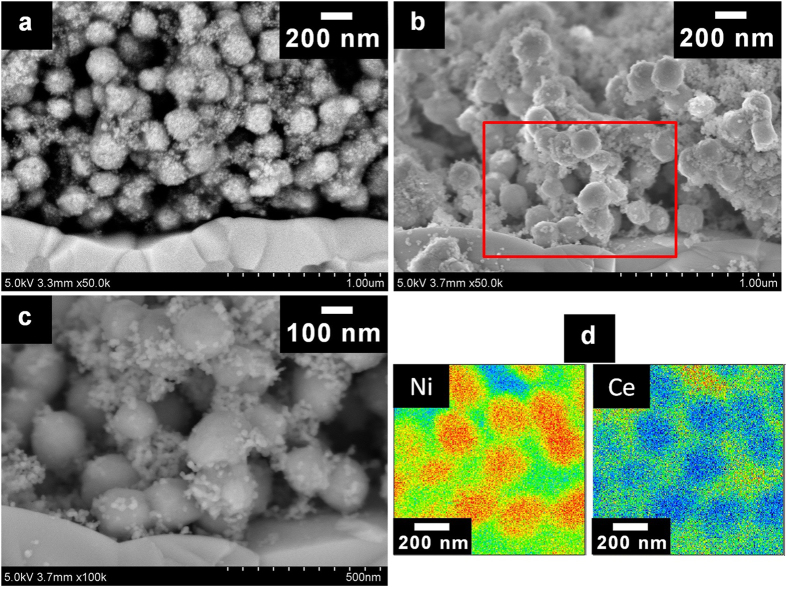
Cross-sectional microscope images of Ni–GDC-nanocube (Ni:GDC = 65:35) anode. (**a**) Back-scattering image (BSI) before power-generation test. (**b**) Secondary-electron image (SEI) after power-generation test operated at 700 °C. (**c**) BSI after power-generation test operated at 700 °C. (**d**) EPMA-WDX mapping images of Ni–GDC-nanocube (Ni:GDC = 65:35) anode after power-generation test operated at 700 °C.

Cross-sectional SEM images of the Ni–GDC-nanocube (Ni:GDC = 65:35) cermet anode before and after the power-generation test at 700 °C are shown in Fig. 3. Before the test, the back-scattering image (BSI) shows that the Ni–GDC-nanocube anode consisted of a composite structure of a metallic Ni sphere covered with fine GDC-nanocube particles (Fig. 3a). Furthermore, it was confirmed that the macrostructure of the Ni–GDC-nanocube cermet as an electrode, which was established before the power-generation test, remained unchanged even after the test at 700 °C (Fig. 3b,c). Meanwhile, the needle-like structures on the surface of the metallic Ni sphere disappeared after the power-generation test. The surface morphology became very smooth (Figs 1a and 3c), which means that the *in situ* fabrication of the metallic Ni framework for electrical power collection resulted from insubstantial crystal growth of the metallic Ni sphere at the operating temperature. The cross-sectional mapping images in Fig. 3d, obtained from electron-probe micro-analysis wavelength-dispersive spectroscopy (EPMA-WDX) of the Ni–GDC-nanocube anode after the power-generation test, indicate that the Ni and Ce signals were clearly separated and formed contrasting images. Furthermore, the core-shell like morphology did not change even after operating at 600 °C for 24 h (Figure S3a and b). It seems that this high stability of novel anode is contributed to the large particle size of Ni spheres and GDC nanocube particles on the surface of Ni spheres. Especially, GDC nanocube particles inhibit the crystal growth and migration of Ni spheres. These results also confirm the high stability of the anode macrostructure.

## Discussion

After analysis of the power-generation properties, the anode material was crushed and its microstructure was closely examined. HR-TEM observations revealed that the initial particle size and {001} crystal facets did not change even after the power-generation test at 700 °C (Figure S4), suggesting that the anode macrostructure and the microstructure of the reaction sites produced during the power-generation test could be preserved. It should be noted that the macro- and microstructure of the anode fabricated without high-temperature sintering showed good power-generation performance and exhibited very high stability during the test.

The anode ohmic resistance (*ηIR*_a_) and polarization resistance (*η*_a_) at 600 °C were evaluated by the current-interruption method (Figure S5). Each *ηIR*_a_ and each *η*_a_ were separated using a Pt reference electrode; the Ni–GDC–nanocube (Ni:GDC = 65:35) anode showed significantly lower *ηIR*_a_ and *η*_a_ than the NiO–GDC anode that was sintered at 1300 °C. During the power-generation test at 500–700 °C, the metallic Ni sphere in the Ni–GDC–nanocube (Ni:GDC = 65:35) anode slightly grew in size, and the macro- and microstructure of the anode remained unchanged. Ni connection was provided by the slight growing up of Ni sphere; therefore, delamination of the anode layer did not occur. It is clear that the enlarged TPB introduced by fine GDC-nanocube particles contributed to the considerable reduction in *η*_a_, which is supported by the SEM images (Fig. 3b–d). On the other hand, it is very interesting that the non-sintered Ni–GDC-nanocube (Ni:GDC = 65:35) anode exhibited obviously lower *ηIR*_a_ than that of the anode sintered at 1300 °C. Electromotive forces using only the metallic Ni sphere as an anode in the power-generation test were present because the contact points between the electrolyte and metallic Ni sphere performed as a TPB. The fine GDC-nanocube particles on the metallic Ni sphere provided many of these contact points, and this enlarged oxygen ion pathway can be regarded as a reason for the considerable reduction in *ηIR*_a_. Therefore, it was suggested that the Ni–GDC-nanocube anode could introduce many ultra-fine contacts to the electrolyte–anode interface as well as the anode functional layer^2^,^3^,^4^.

**Figure 4:**
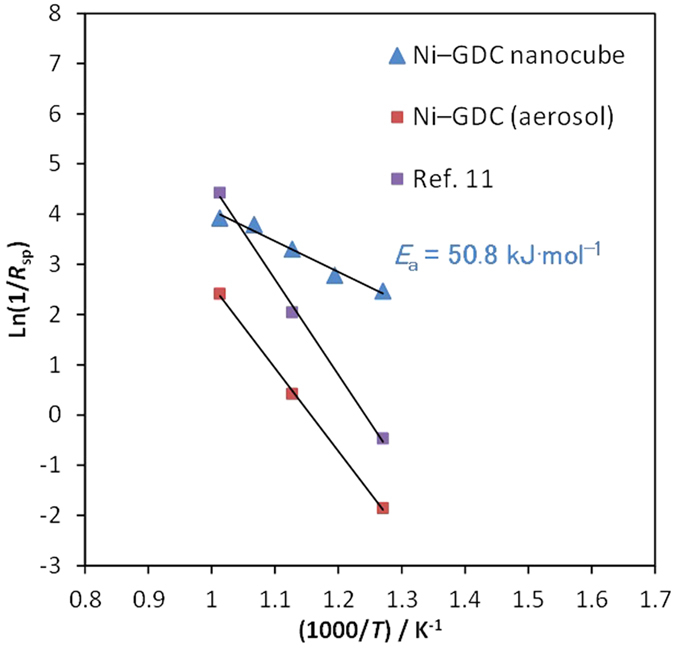
Arrhenius plots of the area-specific resistance (*R*_sp_) of the Ni–GDC-nanocube (Ni:GDC = 65:35) anode at 500–700 °C.

Figure 4 shows Arrhenius plots of the area-specific resistance (ASR or *R*_sp_) of the Ni–GDC-nanocube at 500–700 °C (each impedance spectrum is shown in Figure S6). The activation energy (*E*_a_ = 50.8 kJ mol^–1^) of the Ni–GDC (65:35) nanocube anode is much smaller than those of the Ni–GDC anode sintered at 1300 °C^11^. In this study, the fraction of surface diffusion appeared to be very large compared with anodes sintered at high temperature owing to the use of 10-nm GDC nanocube particles as an oxygen-ion conductor. Therefore, we assume the apparently low activation energy and *R*_sp_ contributed to the increase in reactive {001} facets.

As the GDC nanocube particles were not sintered and did not have contact with each other (Fig. 3b,c, and Figure S3a and b), the allowable oxygen ion pathway length was considered to be very short because of the rather low oxygen-ion conductivity at 600 °C, despite the high power density shown by the single cell with the 20-*μ*m-thick anode. The high power density means that a highly effective electrical pathway was formed along the electrolyte–anode interface to the edge of the anode (electrical collection point). To confirm this supposition, a power-generation test was carried out at 600 °C using a 2-*μ*m-thick anode. We successfully obtained a high power density that was almost the same as that obtained with a 20-*μ*m-thick anode (Figure S7a–c), indirectly supporting the assumption that the oxygen diffusion distance was quite short (<2 *μ*m). These results suggest that novel electrode structure design, which differs from the design for operation at high temperatures, is crucial for low-temperature operation.

We have demonstrated a novel fabrication process of high-performance Ni–GDC-nanocube anode for low-temperature SOFCs, using only a solution-based chemical reaction. The anode material had a core–shell structure, and the core was a metallic Ni sphere that was covered with highly reactive GDC nanocubes. The characteristic composite structure could introduce a much longer electrical pathway (>20 *μ*m) without any sintering. The effective framework of a metallic Ni sphere was formed by means of crystal growth of metallic Ni spheres during the power-generation test at a temperature as low as 500 °C. Meanwhile, the non-sintering fabrication process introduced an enlarged TPB with 10-nm GDC nanocube particles near the interface between the electrolyte and the anode. Such micro- and macrostructure of the anode could considerably reduce *ηIR*_a_ and *η*_a_, and the anode showed great stability even after the power-generation test at 700 °C. We conclude that the Ni–GDC-nanocube cermet anode is a suitable anode material for next-generation low-temperature SOFCs.

## Methods

Details of organic-ligand-assisted hydrothermal synthesis of GDC (Ce_0.9_Gd_0.1_O_1.95_) nanocubes are described in our previous paper^11^. After hydrothermal treatment, the GDC-nanocube precipitates were washed with distilled water and ethanol. The specimen was dispersed in ethylene glycol (EG), and the GDC concentration corresponded to 0.1 M. Similarly, NaOH was also dissolved in EG and the concentration was adjusted to 1 M. NiCl_2_·6H_2_O and the GDC dispersion were added to the EG solvent, and the mixture was heated at 80 °C with stirring. N_2_H_4_·H_2_O was slowly released dropwise into the mixture, while the NaOH solution was added rapidly. After the mixing bar was removed, the mixture was heated at 80 °C for 2 h. The molar ratios of Ni^2+^:N_2_H_4_:NaOH were fixed at 1:20:3. The ratios of other components are summarized in Table S1. After heating, aggregated dark grey or black precipitates were collected with a neodymium magnet and then carefully washed with distilled water and ethanol. The powder was dried at 60 °C for 24 h and mixed with PEG#400 as a binder, and the resulting paste was used as an anode for solid-oxide single-fuel-cell fabrication.

The solid electrolyte comprised a GDC disk that was sintered at 1500 °C for 4 h (thickness: 400 μm; diameter: 15 mm). The La_0.6_Sr_0.4_Co_0.2_Fe_0.8_O_3-δ_ (LSCF) cathode paste prepared by co-precipitation method was deposited by screen printing on the GDC disk, and the coated disk was heated at 850 °C for 2 h. The anode paste was also deposited on the flip side of the GDC disk without post-deposition sintering, and the effective surface area of each electrode was 0.282 cm^2^ (diameter: 6 mm). Power-generation tests were carried out at 500 to 700 °C, following the same procedures described in Ref. 11.

## Additional Information

**How to cite this article**: Yamamoto, K. *et al.* *In situ* fabrication of high-performance Ni-GDC-nanocube core-shell anode for low-temperature solid-oxide fuel cells. *Sci. Rep.* **5**, 17433; doi: 10.1038/srep17433 (2015).

## Supplementary Material

Supplementary Information
