# Supplementary material for: In situ fabrication of high-performance Ni-GDC-nanocube core-shell anode for low-temperature solid-oxide fuel cells
Source: Sci Rep. 2015 Nov 30;5:17433. doi: 10.1038/srep17433 (PMC4663492; doi:10.1038/srep17433)
Supplement: Supplementary Information [file srep17433-s1.pdf]

Supplementary information

***In situ* fabrication of high performance Ni–GDC-nanocube core–shell anode for low-temperature solid-oxide fuel cells**

Kazuhiro Yamamoto*, Nan Qiu and Satoshi Ohara*

Joining and Welding Research Institute, Osaka University, 11-1 Mihogaoka, Ibaraki, Osaka 567-0047, Japan.

Table S1. Fabrication conditions of Ni–GDC-nanocube cermet anode by chemical reduction in a solution heated at 80 °C.

Ni:GDC ratio (v:v)	EG / mL	$NiCl_2 \cdot 6H_2O$ / g	GDC dispersion / mL	$N_2H_4 \cdot H_2O$ / mL	NaOH solution / mL
50 : 50	24.11	0.444	4.94	1.80	5.45
60 : 40	25.76	0.444	3.29	1.80	5.45
65 : 35	26.39	0.444	2.66	1.80	5.45
70 : 30	26.94	0.444	2.12	1.80	5.45
80 : 20	27.82	0.444	1.23	1.80	5.45

The concentrations of the GDC-nanocube dispersion and the NaOH solution were 0.1 and 1 M, respectively. The total volume was adjusted to 36.3 mL.

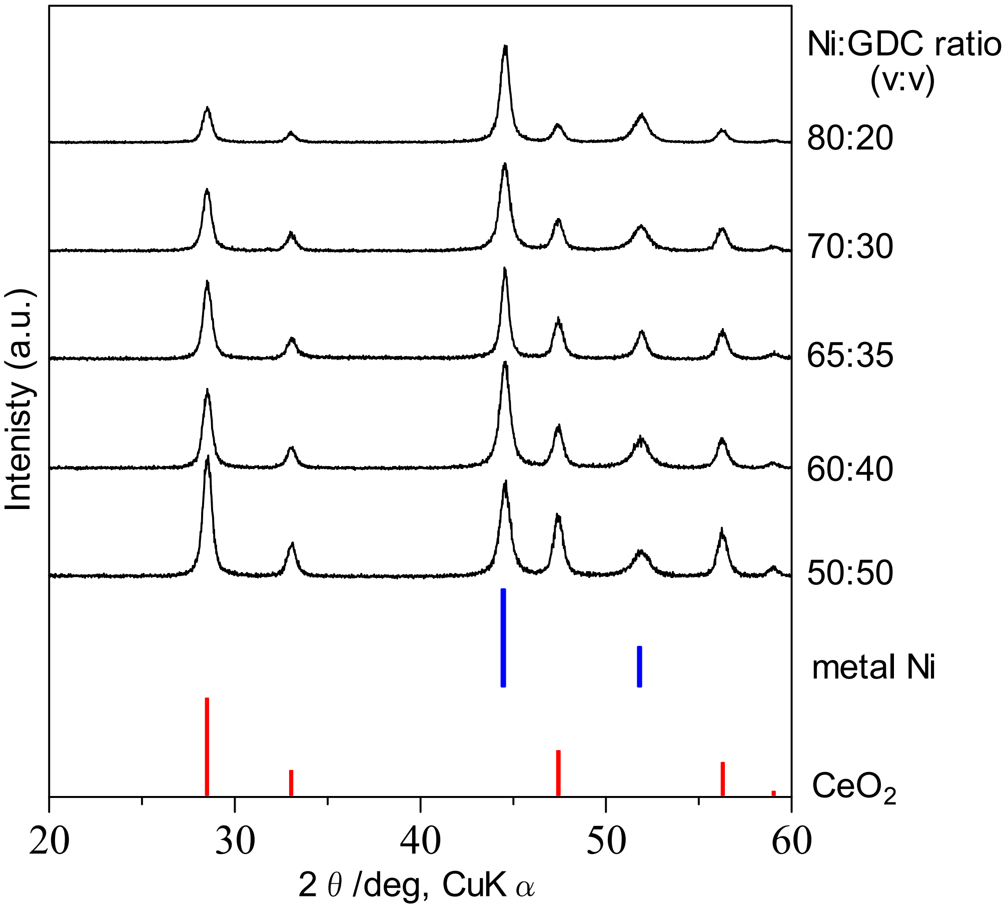

Figure S1. XRD patterns of Ni–GDC-nanocube samples prepared by chemical reduction in a solution heated at 80 °C for 2 h.

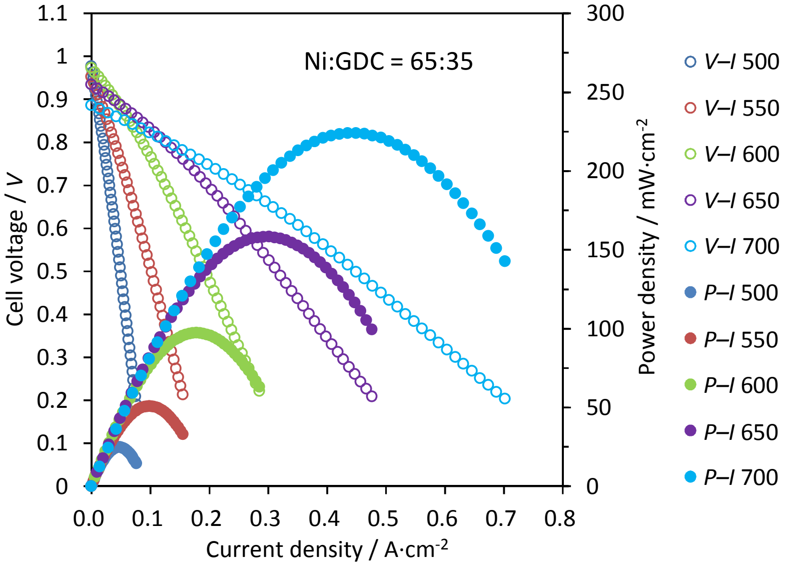

Figure S2. *V–I* and *P–I* curves of SOFC single cell using Ni–GDC-nanocube anode (without any sintering for anode fabrication) operated at 500 to 700 °C.

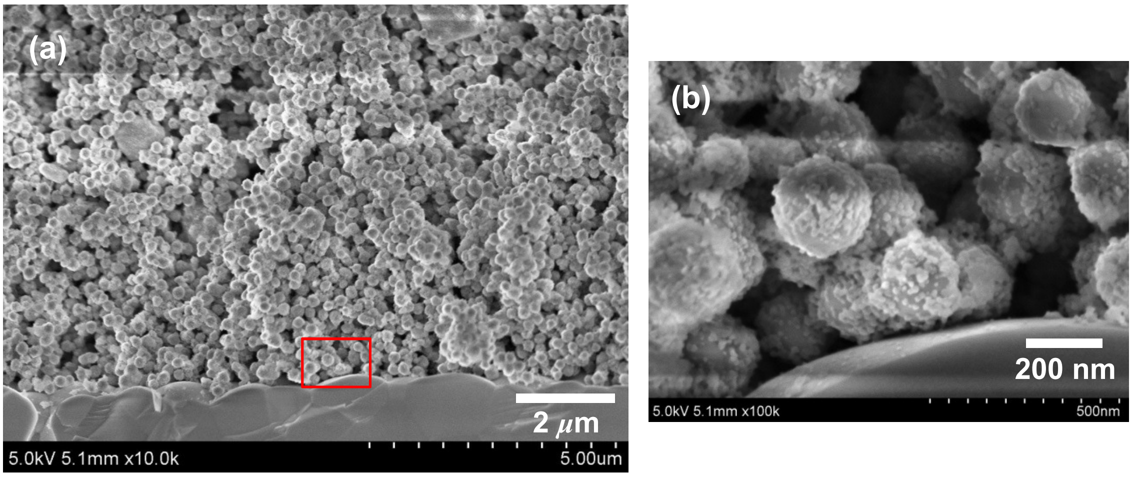

Figure 3S. Cross-sectional microscope images of Ni–GDC-nanocube (Ni:GDC = 65:35) anode after power-generation test operated at 600 °C for 24 h; (a) low-magnification and (b) high magnification.

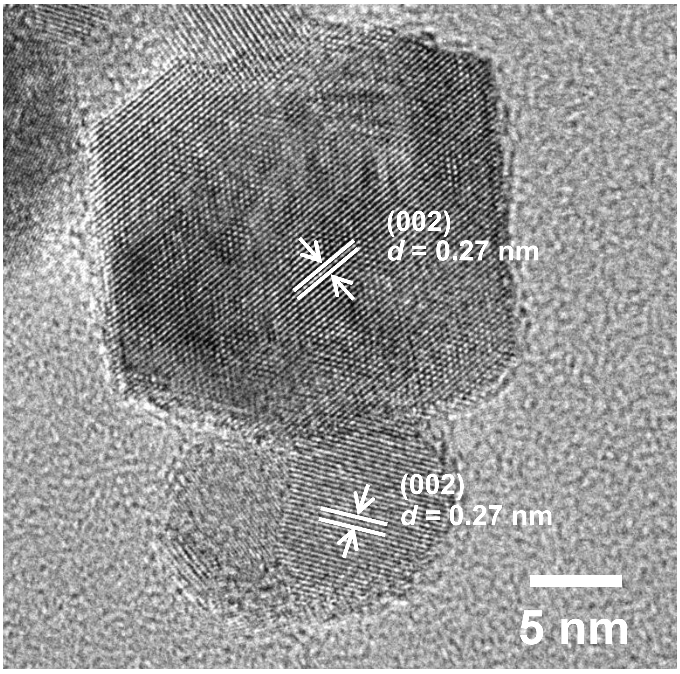

Figure S4. HR-TEM image of GDC nanocubes after power-generation test at 700 °C.

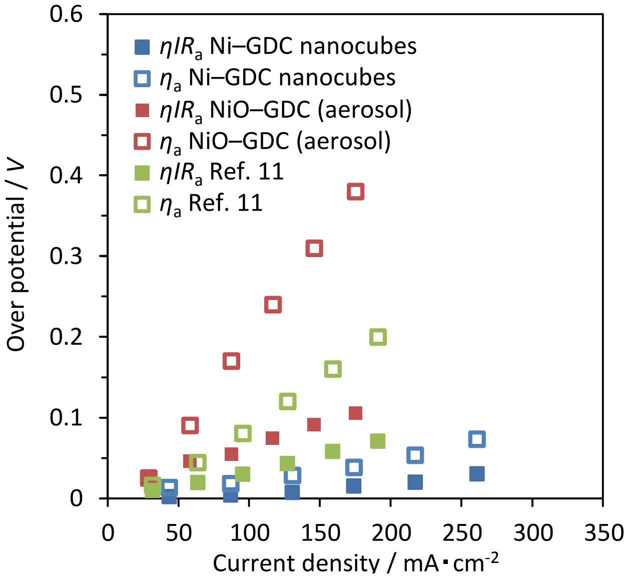

Figure S5. Plots of Ohmic and polarization resistance vs. current density at 600 °C. The data include the results for the Ni–GDC-nanocube (Ni:GDC = 65:35) anode and the Ni–GDC composite anode (aerosol), as well as polarization resistance data from Ref. 11.

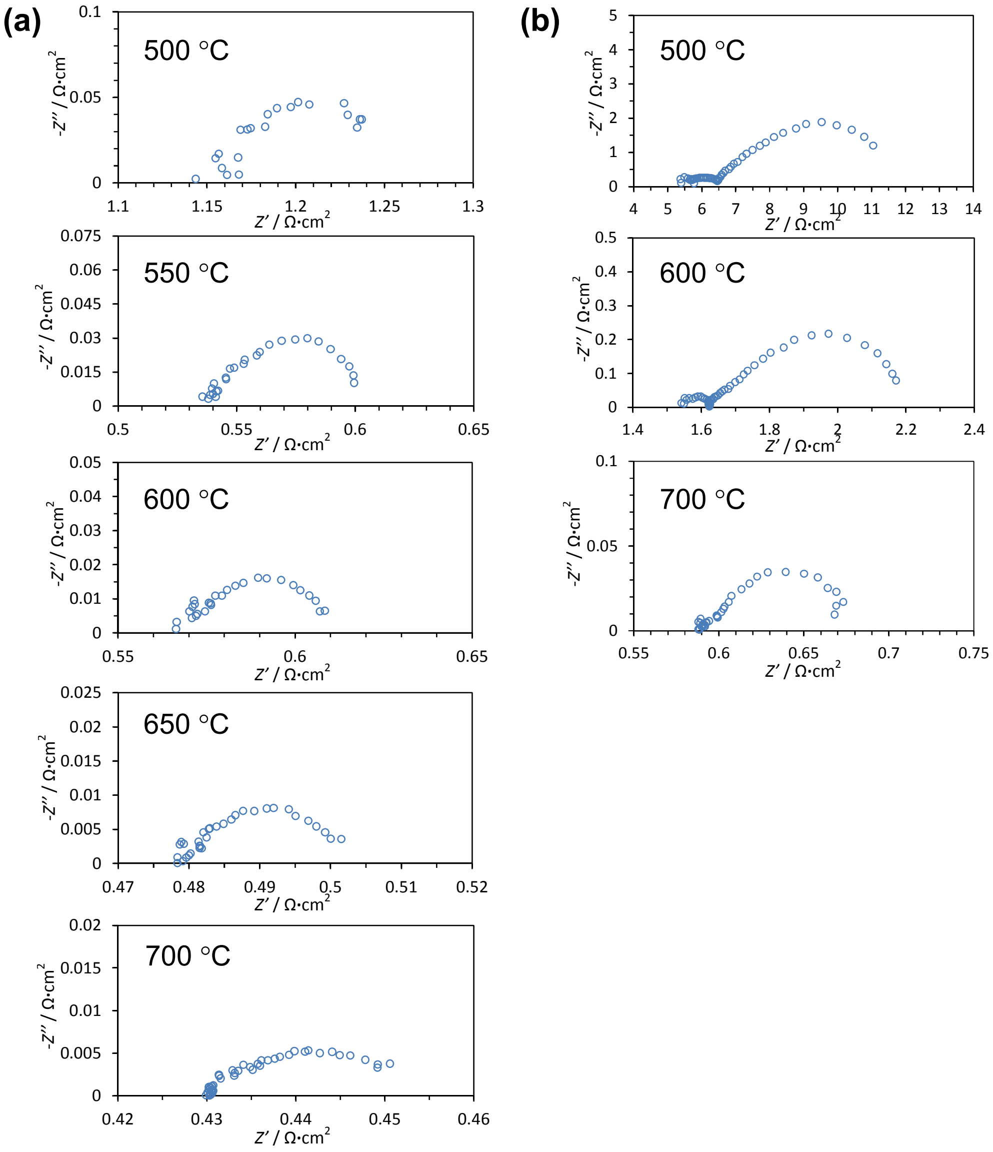

Figure S6. Impedance spectra of various anodes operated at 500 to 700 °C. (a) Ni–GDC-nanocube anode (Ni:GDC = 65:35); (b) Ni–GDC composite anode (aerosol).

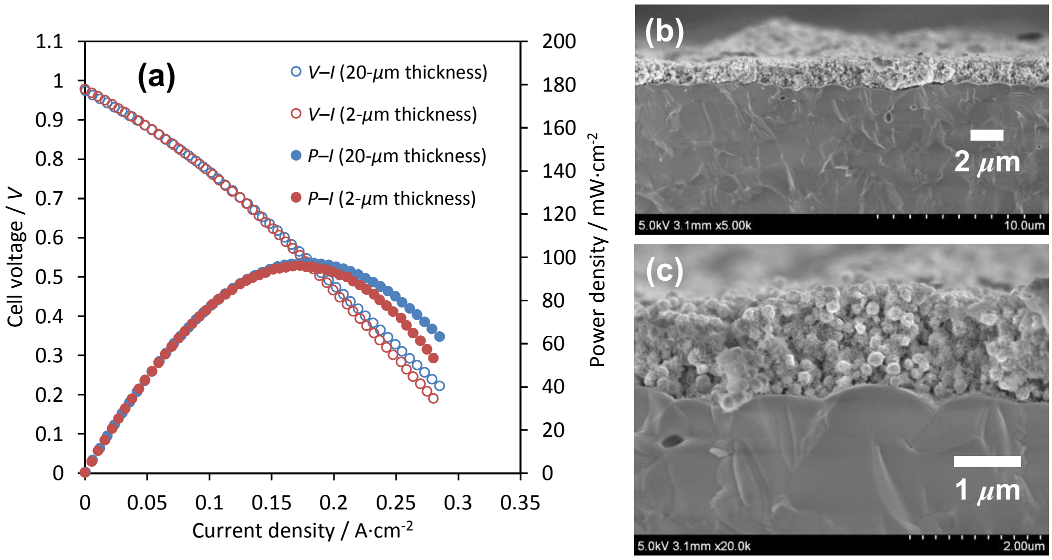

Figure S7. Power-generation tests on Ni–GDC-nanocube anodes. (a) *V–I* and *P–I* curves of single cells using Ni–GDC-nanocube (Ni:GDC = 65:35) anodes with thickness of 2 μm and 20 μm at 600 °C. (b, c) Cross-sectional SEM images of 2-μm-thick Ni–GDC-nanocube anode after power-generation test at 700 °C.
